# Predictive Biomarkers of Immune Checkpoint Inhibitors-Related Toxicities

**DOI:** 10.3389/fimmu.2020.02023

**Published:** 2020-10-06

**Authors:** Ya Xu, Yang Fu, Bo Zhu, Jun Wang, Bicheng Zhang

**Affiliations:** ^1^Cancer Center, Renmin Hospital of Wuhan University, Wuhan, China; ^2^Department of Oncology, Xiangyang Hospital, Hubei University of Chinese Medicine, Xiangyang, China; ^3^Institute of Cancer, Xinqiao Hospital, Army Medical University, Chongqing, China; ^4^Department of Oncology, The First Affiliated Hospital of Shandong First Medical University, Jinan, China

**Keywords:** immune checkpoint inhibitor, toxicity, predictive biomarker, PD-1, PD-L1

## Abstract

The emergence and continuous development of immune checkpoint inhibitors (ICIs) therapy brings a revolution in cancer therapy history, but the major hurdle associated with their usage is the concomitant ICIs-related toxicities that present a challenge for oncologists. The toxicities may involve non-specific symptoms of multiple systems as for the unique mechanism of formation, which are not easily distinguishable from traditional toxicities. A few of these adverse events are self-limiting and readily manageable, but others may limit treatment, cause interruption and need to be treated with methylprednisolone or tumor necrosis factor-α (TNF-α) antibody infliximab, and even directly threaten life. Early accurate recognition and adequate management are critical to the patient's prognosis and overall survival (OS). Several biomarkers such as the expression of programmed cell death ligand 1 (PD-L1), tumor mutation burden (TMB), and microsatellite instability-high (MSI-H)/mismatch repair-deficient (dMMR) have been proved to be the predictors for anti-tumor efficacy of ICIs, but there is a gap in clinical needs for effective biomarkers that predict toxicities and help filter out the patients who may benefit most from these costly therapies while avoiding major risks of toxicities. Here, we summarize several types of risk factors correlated with ICIs-related toxicities to provide a reference for oncologists to predict the occurrence of ICIs-related toxicities resulting in a timely process in clinical practice.

## Introduction

The development of ICIs has changed the systemic treatments of tumors and rewritten history. Even as advanced stage therapy, ICIs have enjoyed unprecedented success in many types of cancers including malignant melanoma (1), non-small cell lung cancer (NSCLC) (2), small cell lung cancer (3), metastatic bladder cancer (4), and urothelial carcinoma (5), etc. Because of such an effective anti-tumor immune response, the Food and Drug Administration (FDA) has approved ICIs for more than thirty indications. With the unprecedented objective response rates (ORR) as well as durable responses across many tumor types, the clinical application of ICIs continues to expand in various combinations including ICIs monotherapy or combination with chemotherapy, radiotherapy, anti-angiogenic agents, or other ICIs.

**Figure 1:**
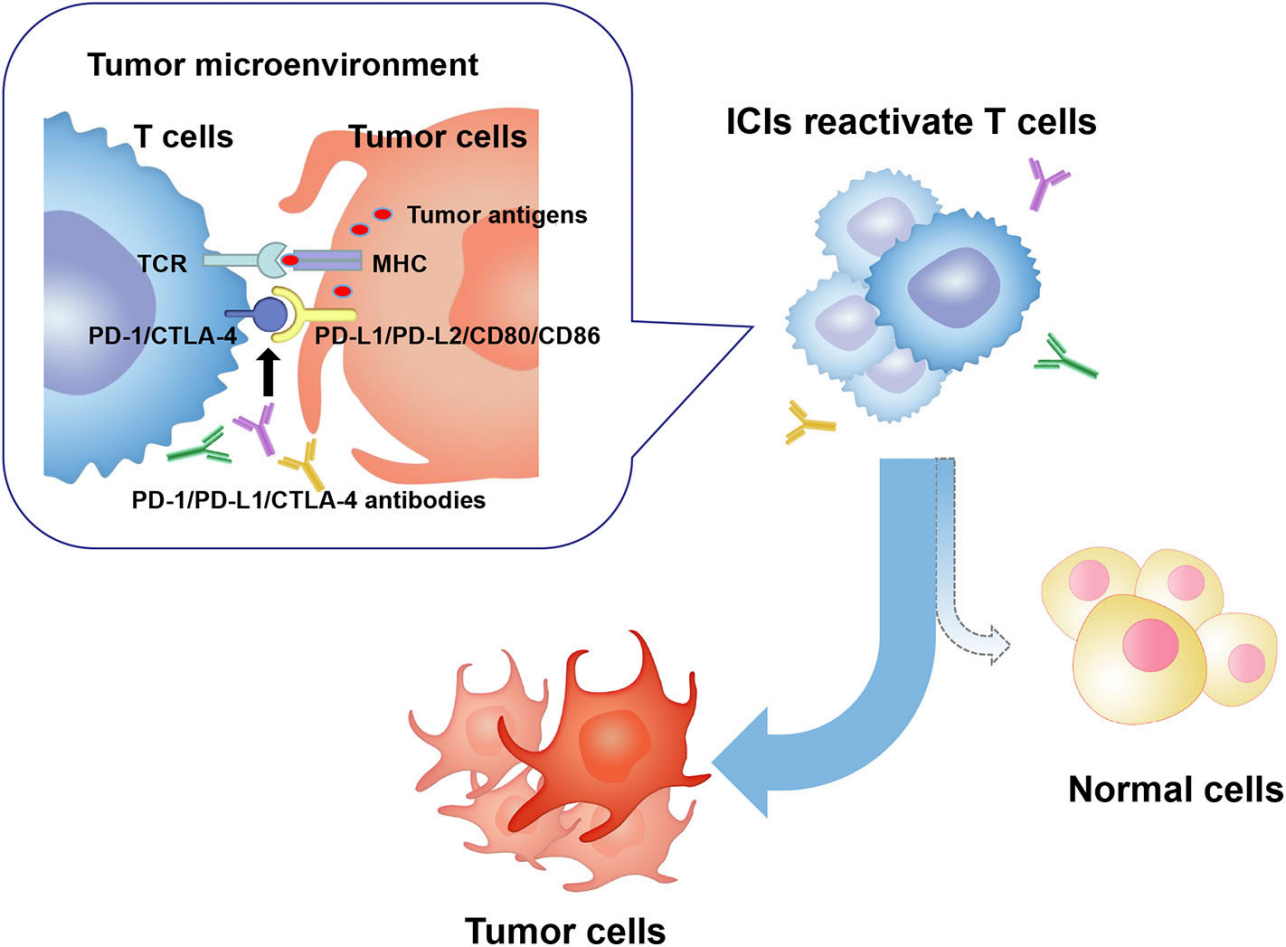
In the tumor microenvironment, PD-1/CTLA-4 molecules on activated T cells are up-regulated and combined to PD-L1/PD-L2 or CD80/CD86 molecules on tumor cells. Consequently, T cell activity is inhibited, and an immunosuppressive microenvironment is formed, which leads to tumor cells escaping the immune surveillance and growing wildly. ICIs restore anti-tumor activity of T cells by targeting and blocking PD-1 or CTLA-4 signaling pathway. Activated T cells kill tumor cells and may attack normal human tissue cells, forming ICIs-related toxicities. PD-1, programmed cell death protein 1; PD-L1/2, programmed cell death ligand 1/2; CTLA-4, cytotoxic T lymphocyte-associated antigen-4; ICIs, immune checkpoint inhibitors; MHC, major histocompatibility complex; TCR, T cell receptor.

ICIs are a novel category of drugs that are essentially humanized monoclonal antibodies, which activate T cells and relieve the immune system to recognize and assault cancer cells by targeting cytotoxic T lymphocyte-associated antigen-4 (CTLA-4) (CD152), programmed cell death protein 1 (PD-1), or programmed cell death ligand 1 (PD-L1). However, the unleashing immune response could increase autoimmunity and cause a plethora of immune-related toxicities, termed ICIs-related toxicities, which can potentially affect any tissue and organs of patients (mainly including gut, skin, endocrine glands, liver, and lung) (Figure 1).

With unclear mechanisms, ICIs-related toxicities may require discontinuation of immunotherapy. Furthermore, because of the early onset and fulminant progression, some severe toxicities are even life-threatening (such as myocarditis, serious colitis, and pneumonia). Oncologists are facing huge challenges in optimizing outcomes during the use of ICIs, which is expected to increase significantly in the years to come. Assuming most toxicities are mild and reversible when detected early and properly managed, searching for predictive biomarkers for the ICIs-related toxicities remain essential for early recognition and appropriate clinical management. Identifying predictive biomarkers to distinguish patients most likely to suffer immune-related adverse events (irAEs) from overall individuals will avoid severe toxicity risk and decrease treatment costs.

**Figure 2:**
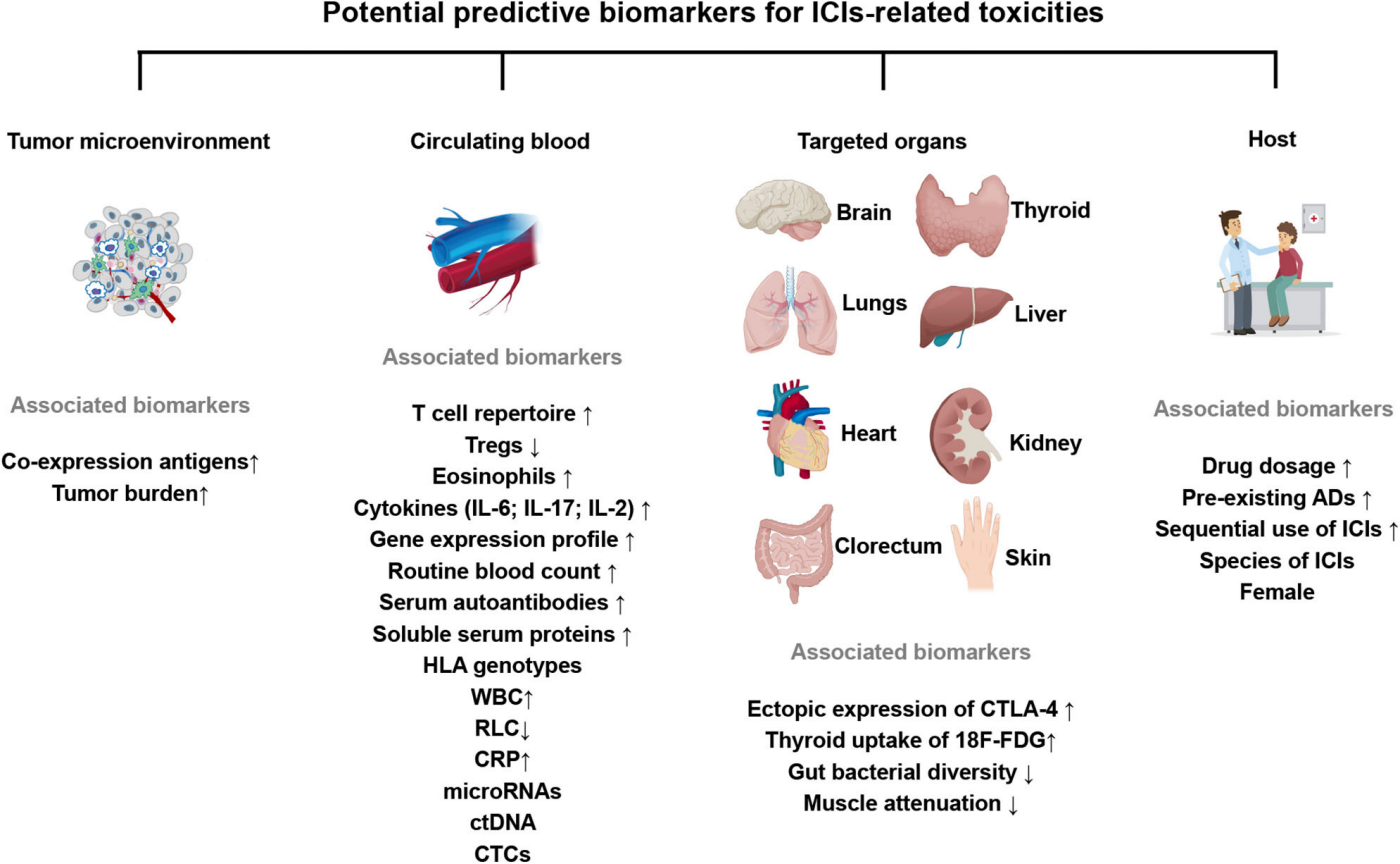
Expression of key potential biomarkers from the tumor microenvironment, circulating blood, target organs or clinical factors, predictive for ICIs-related toxicities. The up or down arrows represent the increase or decrease of biomarkers. All these factors are associated with an increased incidence of ICIs-related toxicities. ICIs, immune checkpoint inhibitors; Tregs, regulatory T cells; IL-6/17/2, Interleukin-6/17/2; HLA, human leukocyte antigen; WBC, white blood cells; RLC, relative lymphocytes count; ctDNA, circulating tumor DNA; CTCs, circulating tumor cells; 18F-FDG, 18F-fluorodeoxyglucose; CRP, C-reactive protein; CTLA-4, cytotoxic T lymphocyte-associated antigen-4; pre-existing ADs, pre-existing autoimmune disorders.

This review focuses on summarizing a variety of potential biomarkers from different sources for ICIs-related toxicities and discussing the unique considerations relevant to patients' treating (Figure 2).

## Predictive Biomarkers for CTLA-4 Inhibitors-Related Toxicities

CTLA-4 plays a pivotal role in inducing peripheral tolerance and maintaining immunologic homeostasis but it is believed to be a negative regulator within the anti-tumor immunity. Specifically, CTLA-4 and CD28 are homologous receptors of T cells, which share a pair of ligands, B7 molecules (CD80 and CD86) expressed on the surface of antigen-presenting cells (APCs) (6). CTLA-4 binds with B7 molecules or detaches them away on APCs in the lymph nodes, causing T cell activation to be inhibited during the primary phase (7). The recombinant fully human immunoglobulin (Ig) monoclonal antibodies, such as ipilimumab and tremelimumab, activate T cells by forming a CTLA-4 pathway blockade at an early stage, of which the former was the first FDA approved inhibitor for unrespectable or metastatic melanoma based on significantly improved survival data in March 2011 (8). However, ICIs-related toxicities were accompanied with the enhanced anti-tumor responses following the CTLA-4 blockade. Ipilimumab-related all grade and 3–4 grade toxicities rates were 86.8 and 28.6%, respectively, which mainly affected gastrointestinal, skin, and renal system (9), while tremelimumab commonly led to gastrointestinal events, dermatologic events, and fatigue (10). Recently, several studies have looked for predictive biomarkers of CTLA-4 inhibitors-related toxicities. Key results are summarized in Table 1.

**Table 1:** Predictive biomarkers for CTLA-4 inhibitor-related toxicities.

**Marker**	**Cancer type (sample size)**	**Source**	**Measurement methods**	**Drugs**	**Correlation**
T cell repertoire (11)	Metastatic prostate cancer (*N* = 27)	Blood	TCR β-chains sequencing	Ipilimumab	Early clonal expansion of CD8 T-cell clones preceded the development of 2-3 CTLA-4-related toxicities
T cell repertoire (12)	Prostate cancer (*N* = 42)	Blood	TCR β-chains sequencing	Ipilimumab	Patients with CTLA-4-related toxicities exhibited greater diversity of CD4^+^ and CD8^+^ T cells
Tregs (13)	Metastatic melanoma (*N* = 26)	Blood	Absolute cell counts	Ipilimumab	A low baseline proportion of peripheral blood CD4+ Tregs was associated with subsequent colitis caused by ipilimumab
Eosinophils (14, 15)	Melanoma (*N* = 156)/(*N* = 43)	Blood	Absolute cell counts	Ipilimumab	The growth count of circulating eosinophils during treatment with ipilimumab was associated with CTLA-4-related toxicities occurrence
Neutrophils (16)	Melanoma (*N* = 115)	Tissue	Biopsy	Ipilimumab	The inflammation of neutrophils was associated with the occurrence of dysregulation of gastrointestinal immunity
IL-6 (17)	Metastatic melanoma (*N* = 140)	Blood	Chemoluminescent immunoenzymatic method	Ipilimumab	Baseline IL-6 serum levels was significantly and independently associated with higher risk of severe toxicity
IL-17 (18)	Metastatic melanoma (*N* = 52)	Blood	Chemoluminescent immunoenzymatic method	Ipilimumab	The fluctuations in blood IL-17 levels was associated with the development and the resolution of colitis symptoms in individually
IL-17 (19)	Metastatic melanoma (*N* = 35)	Blood	Chemoluminescent immunoenzymatic method	Ipilimumab	The baseline serum IL-17 levels were significantly higher in patients with grade 3 diarrhea/colitis
sMICA (20)	Advanced melanoma (*N* = 77)	Serum	ELISA	Ipilimumab	The increased of baseline sMICA related to a lower frequency of ipilimumab-related toxicities
Ectopic expression of CTLA-4 (21)	Advanced melanoma/prostate cancer (*N* = 20)	Tissue	RT-PCR and Western blotting	Ipilimumab	Ectopic expression of CTLA-4 was associated with the onset of hypophysitis
Sex (17)	Metastatic melanoma (*N* = 140)	Clinical characteristics	Logistic regression	Ipilimumab	Female faced higher risk of irAEs
Baseline gut microbiota (13)	Metastatic melanoma (*N* = 26)	Fecal	Next generation metagenomic sequencing	Ipilimumab	Ipilimumab-related colitis was associated with decreased bacterial diversity
Baseline gut microbiota (22)	Metastatic melanoma (*N* = 115)	Fecal	Next generation metagenomic sequencing	Ipilimumab	The bacteroidetes phylum bacteria were more abundant in the faces of the patients who were resistant to ipilimumab-induced colitis
MA (23)	Metastatic melanoma (*N* = 84)	Body composition	CT	Ipilimumab	The decrease of MA significantly associated with high-grade ipilimumab-related toxicities
Pre-existing ADs (24)	Advanced melanoma (*N* = 30)	Clinicopathologic characteristic	Data collection	Ipilimumab	50% of patients with pre-existing ADs experienced exacerbations of their autoimmune or grade 3–5 -CTLA-4-related toxicities

*CTLA-4, cytotoxic T lymphocyte-associated antigen-4; TCR, T cell receptor; Tregs, regulatory T cells; sMICA, soluble major histocompability complex class I chain-related protein A; ELISA, enzyme-linked immunoassay; IL-6/17, interleukin-6/17; MA, muscle attenuation; CT, computed tomography; Pre-existing ADs, pre-existing and early changes in autoimmune disorders*.

### Biomarkers From Circulating Blood

#### T Cell Repertoire

Since CTLA-4 blockade leads to the proliferation of circulating T cells, the diversity of the T cell receptor (TCR) repertoire as well as the expanse of the T cell repertoire are increased simultaneously (25). The clonal expansion of CD8+ T cells occurred predating the onset of grade 2–3 ipilimumab-related toxicities (11), patients with ipilimumab-related toxicities exhibited greater diversity of CD4+ and CD8+ T cells (12), indicating that the early diversification of the T cell repertoire appeared to present with the development of ipilimumab-related toxicities as well as an efficacious prostate-specific antigen responses (12). In total, the checkpoint blockade therapy with subsequent T cell repertoire diversification immediately can be both detrimental and beneficial for patients with cancer, suggesting that oncologists should be more cautious about this indicator.

#### Tregs

Regulatory T cells (Tregs) are a kind of CD4+ T cells that inhibit immunopathology or autoimmune disease *in vivo* by influencing the activity of other cell types. The expression of CTLA-4 on Tregs directly influenced its homeostasis and the function of preventing autoimmunity, the loss of CTLA-4 promoted the expansion of Tregs (6). Zhang's group reported that ipilimumab could prevent CTLA-4 recycling by lysosomal degradation and be less effective in intratumor Tregs depletion and rejection of large established tumors. Notably, the CTLA-4 inactivation led to irAEs (26). The selective depletion of tumor-infiltrating Tregs enhanced by preserving CTLA-4 recycling led to the cancer therapeutic effect of anti-CTLA-4 antibodies (27). That is to say, the depletion of tumor-infiltrating Tregs was closely related to CTLA-4-related toxicities and CTLA-4 molecule inactivation. It was reported that a low baseline proportion of peripheral blood CD4^+^ Tregs was associated with subsequent colitis caused by ipilimumab (13), consistent with previous views that Tregs were capable of suppressing autoimmune diseases (ADs).

#### Eosinophils

A retrospective analysis informed that the growth count of circulating eosinophils during treatment with ipilimumab was associated with ICIs-related toxicity occurrence (14, 15). Furthermore, biopsies of diseased tissue about ipilimumab-associated hepatitis (28), rash (29), and colitis (16) showed the inflammatory infiltrate was similar, and all contained with eosinophils. Similarly, immunohistochemistry revealed the infiltration of CD4+ and CD8+ T cells and highly activated effector cells of affected skin and gut correlated with ipilimumab-related toxicities intensity (30).

#### IL-6

Interleukin (IL)-6 is a pleiotropic inflammatory cytokine acting as a keystone factor in infection, cancer and inflammation. The blocking of immune checkpoints increases cytokine release including IL-6. Notably, low baseline IL-6 serum level was an independent risk factor for ICIs-related toxicities (17). Lower baseline levels of IL-6, IL-8, and sCD25 were associated with subsequent colitis in metastatic melanoma patients treated with ipilimumab (13).

#### IL-17

Compared with no colitis patients, serum IL-17 levels were significantly higher in patients with CTLA-4-related colitis; furthermore, the growth and fall in blood IL-17 levels were, respectively, associated with the development and the resolution of colitis symptoms individually (18). A significant association was demonstrated between baseline circulating IL-17 levels and the later progress of grade 3 diarrhea/colitis after the neoadjuvant treatment of ipilimumab (19). All these studies consistently showed a positive correlation between IL-17 levels and CTLA-4-related toxicities.

#### Other Serum Protein

The release of soluble major histocompability complex class I chain-related protein A (sMICA) compromised natural killer (NK)- cell cytotoxicity, resulting in the tumor's escape from immunosurveillance (31, 32). A visible association was found between a higher baseline serum level of sMICA and lower frequency of ipilimumab-related toxicities (20).

#### Gene Expression Profile

The increased expression of CD177 and CEACAM1 genes, markers of neutrophils activation, were found associated with gastrointestinal toxicity occurrence (33). Similarly, using whole-blood RNA transcript-based models from a169-gene panel, a 16-gene signature (including CARD12, CCL3, CCR3, CXCL1, F5, FAM210B, GADD45A, IL18bp, IL2RA, IL5, IL8, MMP9, PTGS2, SOCS3, TLR9, and UBE2C) was identified to be predictive of tremelimumab-related gastrointestinal toxicities as well as to discriminate patients developing grade 0–1 from grade 2–4 diarrhea/colitis (34).

### Biomarkers From Target Organs

#### Ectopic Expression of CTLA-4

Hypophysitis induced by ipilimumab in about 4% of patients may be attributed to the ectopic expression of CTLA-4 in the pituitary glands, which has been proved at both RNA and protein levels. Furthermore, pituitary antibodies were negative at baseline, increased in the 7 patients with hypophysitis but remained negative in the 13 patients without it (21). In other words, the CTLA-4 molecular expressed ectopically in the pituitary glands or the development of pituitary antibodies may be predictors for the appearance of ipilimumab-related pituitary toxicities.

#### Baseline Gut Microbiota

In recent years, intestinal commensal bacteria has gradually become a popular research direction. Commensal bacteria in the colonic microbiota showed an immunomodulatory effect. For example, the members of Bacteroidetes phylum can limit inflammation by stimulating Tregs differentiation (35). More specifically, colitis was associated with decreased bacterial diversity, the microbiota of patients prone to develop ipilimumab-induced colitis was enriched in Firmicutes at baseline, but others abundant with high proportions of Bacteroidetes phylum in the feces seemed resistant to ipilimumab-induced colitis (13). Besides, a lack of genetic pathways involved in polyamine transport and B vitamin biosynthesis was associated with an increased risk of colitis (22). These affirm the accurately predicted value of the intestinal bacterial spectrum and genome as potential biomarkers for identifying patients who are at risk of developing CTLA-4-related colitis.

#### Muscle Attenuation

With computed tomography (CT), low muscle attenuation (MA) were independent factors significantly associated with high-grade ipilimumab-related toxicities in metastatic melanoma (23).

### Biomarkers From the Host

Valpione et al. (17) found female sex was significantly associated with a higher risk of several ipilimumab-related toxicities, Specifically, abnormal thyroid function happened more frequently in female patients (36).

Compared with 3 and 10 mg/kg dosage of ipilimumab, it showed an ~50% rate of increase of grade 3–5 toxicities and an increased count of toxicities-related death with the higher dosage group (37). Similarly, the incidence level of all grade adverse events with anti-CTLA-4 treatment was 61% for 3 mg/kg dosage and 79% for 10 mg/kg (38). These dose effects corroborated that the dosage decided the risk of developing ipilimumab-related adverse events.

## Predictive Biomarkers for PD-1/PD-L1 Inhibitors-Related Toxicities

PD-1 molecule is an inhibitory receptor that was expressed on activated T cells and avoids unwanted inflammation and tissue damage caused by the excessive activation of T cells. But tumor cells take advantage of immune-tolerance mechanisms by up-regulating the expression of PD-1 ligands, PD-L1 (B7-H1), and PD-L2 (B7-DC). Subsequently, the binding of PD-1 and its ligands in the peripheral tissues inhibits those already activated T cells in the immune response. The production of monoclonal antibodies target the PD-1/PD-L1 signaling pathway to mobilize the host autoimmune system's anti-tumor potential. PD-1/PD-L1 monoclonal antibodies significantly improve the survival of patients with advanced malignancies compared to chemotherapy, and they are now being used as the second-line, or even first-line treatment in many types of cancers. However, it can also provoke powerful autoimmune reactions in other organ systems, classified as PD-1/PD-L1 inhibitors-related toxicities. PD-1-related serious adverse events were reported to occur at a percentage of 11% with a 1% rate of PD-1-related deaths (39). Key results about predictive markers for PD-1/PD-L1 inhibitors-related toxicities are summarized in Table 2.

**Table 2:** Predictive biomarkers for PD-1/PD-L1 inhibitors-related toxicities.

**Marker**	**Cancer type (smple size)**	**Source**	**Measurement methods**	**Drugs**	**Correlation**
Routine blood count (40)	Melanoma (*N* = 101)	Blood	The count of blood cells	Nivolumab	An increased WBC count and decreased RLC were independently associated with lung/gastrointestinal toxicities
TgAbs and TPOAbs (41)	cancer (*N* = 66)	Blood	TOSOH	Nivolumab	The baseline TgAbs and TPOAbs levels were associated with the development of thyroiditis
TgAbs and TPOAbs (42)	Advanced NSCLC (*N* = 137)	Blood	TOSOH	PD-1 inhibitors	Thyroid dysfunction was more frequent among patients with pre-existing antithyroid antibodies
Rheumatoid factor (42)	Advanced NSCLC (*N* = 137)	Blood	TOSOH	PD-1 inhibitors	Skin reactions were more frequent among patients with pre-existing rheumatoid factor
TgAbs and TPOAbs (43)	Advanced malignant diseases (*N* = 26)	Blood	TOSOH	PD-1 inhibitors	The pre-existing and early increase (≤4 weeks) in TgAbs and TPOAbs levels were found to be associated with PD-1-related thyroid toxicities
Tg (43)	Advanced malignant diseases (*N* = 26)	Blood	Chemiluminescence immunoassay	PD-1 inhibitors	The early increase in Tg levels were strongly associated with development of thyroid irAEs
sCD163 and CXCL5 (44)	Advanced melanoma (*N* = 46)	Blood	ELISA	Nivolumab	The serum absolute level of serum sCD163 was significantly increased in patients with nivolumab-related toxicities, accompaning by an increasing trend of CXCL5
Pre-existing ADs (45)	Advanced melanoma (*N* = 119)	Clinicopathologic characteristic	Data collection	PD-1 inhibitors	20 (38%) patients had a flare of ADs requiring immunosuppression, 2 (3%) patients experienced a recurrence toxicities the same with ipilimumab while 23 (34%) developed new toxicities
Pre-existing ADs (46)	NSCLC(*N* = 56)	Clinicopathologic characteristic	Data collection	PD-(L)1 inhibitors	55% patients developed an ADs flare and/or ICIs-related toxicities
Pre-existing ICIs-related toxicities (47)	Metastatic melanoma (*N* = 80)	Clinical characteristic	Data collection	PD-1inhibitors	31 patients experienced clinically significant recurrent or distinct toxicities

*WBC, white blood cells; RLC, relative lymphocytes count; TgAbs, anti-thyroglobulin antibodies; TPOAbs, anti-thyroid peroxidase antibodies; sCD163, soluble CD163; PD-1, programmed cell death protein 1; PD-L1, programmed cell death-ligand 1; NSCLC, non-small-cell lung cancer; ICIs, immune checkpoint inhibitors; Tg, thyroglobulin; ADs, autoimmune diseases; TOSOH, electrochemiluminescent immunoassay; ELISA, enzyme-linked immunosorbent assay; irAEs, immune related adverse events*.

### Biomarkers From Circulating Blood

#### Routine Blood Count

The routine blood count is a basic and routine examination for clinical tumor inpatients. After univariate analysis and multivariate analysis in routine blood count data, an increased white blood cells (WBC) count and decreased relative lymphocytes count (RLC) were independent factors associated with lung/gastrointestinal toxicities (40). The baseline Absolute eosinophils count >240/μL or relative eosinophils count could be useful biomarkers to predict PD-1-related endocrine toxicities (48). Besides, numerous neutrophils infiltrated into the skin of one of the nivolumab-associated psoriasiform dermatitis patients (49). It is manifested that these factors could be a prompting signal of PD-1-related toxicity occurrence.

#### Th1

CD4+ helper T cells (Th) 1 are key regulators in the tumor immune microenvironment and have a crucial role in activating cytotoxic T lymphocytes, participating in the pathological response process of inflammatory bowel disease (IBD) and rheumatoid arthritis (RA). Previous study showed that the subepithelial layer was enriched with CD4+ T cells in colitis induced by CTLA-4 inhibitors (50). The increased numbers of Th1 in tumors was reported to be associated with an improved response to immune therapies (51). By contrast, the presence of high CD4+ and low CD8+ tumor-infiltrating lymphocyte levels were independent predictors of poor progression-free survival (PFS), while the former was positively correlated with late tumor stage (52). Infiltration of Th1 in the colon suggested that the development of nivolumab-related colitis is associated with Th1 dominant response (53).

#### Serum Autoantibodies

Hypothyroidism was observed in 8.6% of metastatic melanoma patients treated with nivolumab (54), thyroid dysfunction has been reported to be one of the most frequent nivolumab-related adverse events. Compared with those patients free of thyroiditis, the baseline anti-thyroglobulin antibodies (TgAbs) and anti-thyroid peroxidase antibodies (TPOAbs) levels were significantly higher in destructive thyroiditis patients (41), the appearance of thyroid dysfunction during PD-1 treatment closely associated with anti-thyroid antibodies (55). Toi et al. (42) assessed the relationship between the safety and efficacy of anti-PD-1 treatment and preexisting autoimmune markers, found that the clinical outcomes, including PFS, ORR, and disease control rate, were significantly better among patients with any of the preexisting antibodies positive. Moreover, thyroid dysfunction was more frequent among patients with preexisting thyroid autoantibodies (TgAbs and TPOAbs) (20 vs. 1%, *P* < 0.001) while skin toxicities were more frequent among patients with preexisting rheumatoid factors (47 vs. 24%, *P* = 0.02). In addition, the pre-existing and early increasing (≤4 weeks) serum thyroid autoantibodies levels were found to be associated with ICIs-related thyroid toxicities (43). The homology of tumor-associated antigen NY-ESO-1 with thyroid autoantigens leads to the cross presentation, which might partly explain the mechanisms of PD-1/PD-L1 related thyroid toxicities (56).

PD-1/PD-L1 related autoimmune diabetes (type 1 diabetes mellitus, T1DM) were rare with an incidence of 1% ~53% of which had at least one positive islet autoantibody (57) and 21% had two or more (58). A Hispanic boy with insulin autoantibody and islet antigen 2 antibody-positive, was reported to suffer from T1DM which presented with acute progression to hyperglycemia and diabetic ketoacidosis after treated with pembrolizumab because of the progression of classical Hodgkin lymphoma (59). Briefly, the autoantibodies would be great potential predicting indicators for these endocrine toxicities with genetic disposition, which need to be detected before ICIs to assess risk.

#### Soluble Serum Proteins

Immune-mediated myocarditis was rare but presented unique clinical challenges due to non-specific presentation, exclusive diagnosis, and potentially life-threatening consequences, and the time-critical need to differentiate it from other causes of cardiac dysfunction. In some cases, the common cardiotoxicity markers, troponins, and BNP were found to be raised (60, 61), circulating anti-conductive tissue autoantibodies (ACTA) was suggested as a possible biomarker (62). The true incidence of ICIs-included cardiotoxicity is presently unknown, the biomarkers are needed for early identification and diagnosis of myocarditis because of the fatal consequences. Since the serum absolute levels of serum soluble CD163 (sCD163) and CXCL5 were significantly increased in patients who developed nivolumab-related toxicities, the absolute level of sCD163 and CXCL5 may serve as possible prognostic biomarkers (44). Besides, low serum albumin was reported as an independent risk factor for PD-1-related pneumonitis (63). The baseline and early increase (before 4 weeks) in serum thyroglobulin (Tg) levels were strongly associated with the development of thyroid irAEs (43). C-reactive protein (CRP) level and IL-6 were observed to reflect the clinical course of colitis clearly, which exposed the potentiality nivolumab-related toxicities predictive value (53).

#### HLA Genotypes

As we know, the human leukocyte antigen (HLA) genotypes are strongly associated with many kinds of autoimmune diseases. For example, the HLA B27 was identified as the susceptibility gene of ankylosing spondylitis (AS); HLA-DR3 was reported as the main predisposing allele for autoimmune thyroid diseases (64). Susceptible HLA genotypes dominated by DR4 were present in 76% patients with PD-1/PD-L1 related T1DM (58). The dominance of susceptible HLA genotypes indicates the potentiality in identifying patients who are at the highest risk of suffering from T1DM during ICIs treatment.

### Biomarkers From the Host

Similarly to CTLA-4, compared with men, women were more likely to develop all grades of PD-1-related toxicities (mainly including pneumonitis and endocrinopathies). Interestingly, endocrinopathies were more common in premenopausal women than postmenopausal women or men (67 vs. 60 vs. 46%) (65). The female sex is known as one of the risk factors for autoimmune diseases, which provides us with an idea that differences in sex hormone levels in patients may affect the incidence of toxicity. It reminds us that in the era of precision treatment, we need to record the clinical characteristics and baseline hormone level status of patients before treatment in more detail.

### Other Biomarkers

Because of the non-invasive, intuitive, and fast advantages, imaging examination plays an irreplaceable role for cancer patients in diagnosing, staging, curative effect evaluation, and adverse events detection in clinical practice. With the 18F-fluorodeoxyglucose (18F-FDG) positron emission tomography-computed tomography (PET/CT), thyroid gland diffuse increased 18F-FDG uptake was observed in majority of patients at the period of PD-1-related thyroid toxicities suffering (45, 46). the baseline thyroid uptake of 18F-FDG increased the risk of nivolumab induced thyroid toxicity development (47). Therefore, with dynamic imaging monitoring, the dynamic changes of thyroid 18F-FDG uptake may predict PD-1 related markers of thyroid toxicity. Additionally, with chest CT, the baseline fibrosis score ≥1 (0–5) was the only risk factor for PD-1-related pneumonitis (66).

## Combined Predictive Biomarkers

Because of the similar onset mechanism, there were some overlaps between PD-1, PD-L1, and CTLA-4 blockades related toxicity spectra, indicating the presence of common predictors.

### Biomarkers From Circulating Blood

#### Circulating Blood Cells

Since the elevated baseline neutrophil-to-lymphocyte ratio (NLR) and absolute neutrophil counts were significantly correlated with poor outcome data of immunotherapies, a neutrophil-based index was suggested as biomarkers for risk-group stratification (67). Neutrophils are the main components of inflammatory infiltration; higher-grade colitis was associated with endoscopic inflammation (68). Lamina propria infiltration by neutrophils was associated with the occurrence of dysregulation of gastrointestinal immunity after the CTLA-4 was blocked (16). Low NLR and low platelet-to-lymphocyte ratio at baseline were confirmed as independent predictive markers of the development of ICIs-related toxicities (69). Besides, early changes in B cells induced by inhibitors combined-treatment predicted higher rates of higher-grade ICIs-related-toxicities after therapy (70). Taken together these findings suggest a potential predictive role of circulating blood cells as markers for ICIs-related-toxicities development in a category of patients, which is easy to measure in daily practice.

#### Serum Pro-inflammatory Cytokines

Blood markers, such as the raised serum levels of lactate dehydrogenase and CRP were identified as risk factors for poor survival in patients treated with ICIs (71). In view of the finding of 11 significantly upregulated cytokines (including proinflammatory cytokines such as IL-1α, IL-2, and IFN-α2) in patients with severe toxicities at baseline and early during ICIs treatment, these were integrated into a single toxicity score and validated to predict for high grade ICIs-related toxicities in patients treated with combination immunotherapy (72). In addition, a significant serum IL-6 levels increase in patients with psoriasis-afflicted or other ICIs-related toxicities after nivolumab treatment while decreases were observed in non-afflicted metastatic melanoma patients (49).

Anti-CTLA-4 antibodies could regulate the unfolding of autoimmune diabetes (73). PD-1/PD-L1 binding played an important role in preventing the onset of diabetes in mouse models (74). The existence of PD-1 or CTLA-4 genetic polymorphisms in humans was linked to series of autoimmune diseases (ADs) susceptibility, which mainly included RA, AS, T1DM, and graves' disease. For different genotypes, the correlation with ADs was inconsistent (75–77). Indeed, ICIs treatment can cause or exacerbate ADs including T1DM (78), Immune checkpoint-associated gene polymorphisms may be potential predictors of ICIs-related autoimmune toxicity, but still need to be validated in clinical practice.

### Biomarkers From Tumor Microenvironment

#### Co-expression Antigens

A case report which made the post-mortem evaluation and analyzed the immune infiltrated tissues (including skeletal muscle and myocardium) and tumor in two melanoma patients suffered from fulminant myocarditis after the combination treatment with ipilimumab and nivolumab, presented the most abundant TCR type increases in one of the patients. Additionally, tumors in the two patients expressed abundant desmin and troponin which belonged to the muscle-specific antigen (61). These may be interpreted that the presence of common antigens between tumor and healthy tissue caused the myocarditis, supporting with recent views that the cross-presentation of shared antigens might lead to autoimmunity in patients treated with ICIs.

#### Tumor Burden

According to the Response Evaluation Criteria in Solid Tumors version 1.1 (RECST 1.1), tumor burden was defined as the sum of the longest diameters for a maximum of five target lesions and up to two lesions per organ and accessed by CT (79). A higher tumor burden was a significant independent predictor of severe irAEs (*P* = 0.03) (80) and poorer survival (*P* < 0.01) (79).

### Biomarkers From the Host

ICIs may trigger a higher risk of toxicity among patients with pre-existing autoimmune disorders (ADs) or inflammatory diseases, which excluded such patients from most clinical trials involving ICIs therapy and the relevant data were limited (24). These situations led to a gap in clinical needs for those patients. Patients with pre-existing ADs (including RA, psoriasis, IBD, systemic lupus erythematosus, multiple sclerosis, and autoimmune thyroiditis) treated with ipilimumab, 50% of those experienced exacerbations of their autoimmune or grade 3–5 ICIs-related toxicities (24). Then, after identifying another 119 melanoma patients with pre-existing ADs and/or ipilimumab-related adverse events treated with PD-1 blockades, 38% patients experienced a flare of ADs requiring immunosuppression, 3% suffered a recurrence toxicity while 34% developed new toxicities (81). A 55% rate of patients developed an ADs flare and/or an ICIs-related toxicity in PD-1/PD-L1 antibodies treated NSCLC patients with ADs (82). A serious case of metastatic melanoma patients resumed PD-1 therapy after suffering from combination ICIs treated-related toxicities, ultimately, 39% of patients experienced clinically significant recurrent or distinct toxicities (83). Recently, multivariable analyses showed whether the pre-existing ADs identified by strict criteria or relaxed criteria were both associated with the ICIs-related toxicities diagnosis during hospitalization therapy resumption (84). These results consistently indicated that pre-existing ADs may be a predictor of toxicity. Notably, ICIs could be considered in this setting with vigilant clinical monitoring after a detailed and comprehensive assessment on the risk vs. benefit for each case.

As a matter of fact, ICIs-related toxicities in different tumor types showed a regular pattern. For instance, gastrointestinal and skin toxicities were more common in melanoma patients. Compared with melanoma, NSCLC had a higher rate of pneumonitis. Arthritis and myalgia happened more frequently in melanoma patients compared to renal cell carcinoma where pneumonitis and dyspnoea were more endemic (85).

Generally, PD-1-related toxicities were different from CTLA-4. Compared with PD-1 inhibitors, more high-grade toxicities occurred in patients treated with CTLA-4 inhibitors (86). Specifically, all grades colitis, hypophysis, and rash were more common with CTLA-4 inhibitors, whereas PD-1 inhibitors had increased risk for development of pneumonitis, hypothyroidism, arthralgia, and vitiligo (85). There was a greater risk of hyperthyroidism in patients with PD-1 inhibitors than PD-L1 inhibitors. Even as for PD-1 inhibitors, the rates of hyperthyroidism were significantly different between nivolumab and pembrolizumab (87). That is to say, the species of ICIs are closely related to the occurrence of different toxicities. For patients with different basic conditions, the choice of ICIs is important to the overall efficacy and safety of the patient.

Combination strategies of ICIs have been suggested to expose synergistic effects on the activation of anti-tumor immune response and increase the response rates in patients, which may offer promising future cancer treatments (88–90). Because of immunotherapy resistance or toxicity, sequential therapy with two or more ICIs to prolong survival in cancer patients is becoming more common in clinical practice (91). However, ICIs-based combination therapy leads to a relatively high incidence of ICIs-related toxicities coexisting with improved efficacy (92, 93). Severe ICIs-related pneumonitis (94), fulminant cardiotoxicities (95) or other severe toxicities (91) were observed in lung cancer patients re-treated with PD-(L)1 inhibitors after having experienced previous ICIs treatment, indicating that the sequential and combined use of ICIs treatment for patients may predict higher frequency of toxicity. Likewise, the incidence of ICIs-related pneumonitis was marginally higher in those lung cancer patients who received prior chest radiotherapy than patients who did not (96); Curative-intent chest radiotherapy may increase the risk of any grade ICIs-related pneumonitis (97). Therefore, great caution is needed in patients receiving ICIs in combination or sequel.

## Discussions

Immunotherapy represents a major breakthrough for several cancers, but only 20–30% of patients with malignancies respond to ICIs. Unfortunately, the incidence of any-grade irAEs is more than 50%, including a significant proportion of serious and occasionally life-threatening irAEs, and treatment-related deaths occur in up to 2% of patients (98). Based on this, tumor immunotherapy requires the selection of the most beneficial population based on minimizing the risk of irAEs. Looking for highly efficient and specific predictive biomarkers is an urgent problem during the current stage of the explosive application of immunotherapy. Identification and investigation of potential biomarkers that may predict the development of ICIs-related toxicities are areas of active research. Several potential biomarkers have been reported to show the early predicative value of ICIs-related toxicities, which mainly taken from circulating blood, affected organs, tumor microenvironment, and clinical parameters.

Generally, biomarkers from affected organs or tumor microenvironments requires tissue biopsy, which is useful for predicting the biological behavior, especially for high heterogeneous tumor tissue (99). Besides, histopathological and immunohistochemical are mature clinical routine examination methods. However, tumor and immune microenvironments may change dynamically during tumor development and treatment. It is necessary to dynamically observe biomarkers to accurately reflect the actual state at different time points which requires repeated biopsy, but the invasive procedures are inevitable steps which are not allowed to be used often and may cause additional side effects such as infection. In these subtypes, the gut microbiota is an exceptional biomarker, as it can be collected from the patient's feces without intrusive steps. Therefore, it has unique clinical application value in this view and needs further verification in prospective studies. Compared with biopsy samples from tumor tissue, circulating blood sample is more available because of negligible invasion, it is an ideal access to monitor the shift of biomarkers in peripheral blood for predicting the development of ICIs-related toxicities. Circulating blood-based liquid biopsy holds a high position in oncology because of the unique advantages and wide clinical applications, such as estimating overall tumor heterogeneity, dynamic tracking temporal-based tumor heterogeneity, and assessing response to therapy early in real time (100). In the age of precision medicine, with the development of cutting-edge molecular diagnostics, soluble immune checkpoint molecule, exosomal protein, exosomal microRNA, circulating tumor cells (CTCs), and circulating tumor DNA (ctDNA) were established to be closely correlated with tumor diagnosis, staging, monitoring, and prognosis (100–105). These molecules played critical roles in tumorigenesis and tumor progression; therefore, they demonstrated a promising predictive value at the clinical treatment efficacy and ICIs-related adverse events. Advances in technologies such as sequencing will bring more translational research and clear mechanisms of action, making the treatment of cancer patients more personalized and efficient. For example, the pre-exciting thyroid autoantibodies were significantly associated with subsequent PD-1 related toxicities and with the clinical benefits (42). Some other potential clinical factors predictive for severe ICIs-related toxicities have also been proposed, including family history of ADs, tumors infiltration and location, previous viral infections (HIV or hepatitis B virus) and the concomitant use of medicines with known autoimmune toxicities (106, 107). Additionally, monitoring muscle attenuation, tumor burden and thyroid 18F-FDG uptake through imaging examinations is also the preferred solution, because it is relatively convenient, quick and non-invasive. From a practical clinical perspective, these data are relatively simple to collect and do not require additional financial burden on patients, most biomarkers are practical.

The oncologists should be familiar with every patient in detailed medical history and basic conditions (included but not limited to routine blood counts, lymphocyte typing, cytokine and autoantibody detection, and gender, age, basic immune status, and other general situation) before immunotherapy, watch out for any new or worsening symptoms as well as detailed and dynamic but comprehensive auxiliary inspection during the treatment, evaluate it in time and treat these toxicities. For example, tocilizumab can target inhibit IL-6 in order to prevent the increased of IL-6 and stop related toxicities (108); patients with high titer of TgAbs and TPOAbs who developed to grade 2 hypothyroidisms early take a moderate dosage of L-T4 therapy throughout (43). Then, a close follow up is also necessary after treatment.

Identification of biomarkers that predict the onset of ICIs-related toxicities has great relevance in clinical practice, as it could help identify patients earlier that are particularly susceptible to distinct forms of immunotherapy-induced adverse events, and consequently facilitate proper preemptive management and not only reduce the risk of severe toxicities, discontinuation of medication or obstruction of efficacy but also the costs of treatment. Since a single biomarker change is often related to a certain type of toxicity, it is necessary to increase the types of biomarker and combine them, with dynamic and continuous monitoring, in order to comprehensively analyze the risk of toxicity in patients and perform treatment, benefit in survival but also improve the quality of life on the premise of maximization.

In conclusion, we summarized multiple kinds of potential biomarkers and discussed their respective advantages and disadvantages. Firstly, additional studies are still needed to confirm the predictive value of potential biomarkers and identify other risk factors for irAEs to ICIs helping to determine the patients who are able to maximize the therapeutic benefits while minimizing irAEs. Secondly, with the changes of the immune microenvironment and tumor status in tumor patients at different stages, most biomarkers require dynamic monitoring and combined analyzing so as to predict the possible risks and development direction more comprehensively and accurately and deal with them in time. Thirdly, irAEs predictive biomarkers are still in the exploratory stage recently. We insist that oncologists should examine different kinds of potential biomarkers as comprehensively as possible in clinical practice, to comprehensively assess the risk-benefit ratio for individual patients and maximize therapeutic benefits while minimizing irAEs. In the future, the focus should perhaps be on effective screening of the benefit-seeking population through the diversified detection based on liquid biopsy and the combination of new immune toxicities related biomarkers. Furthermore, precision strategy should be applied in irAE management according to patient's toxicity features associated with cellular and molecular mechanisms including T cell activation, and inflammatory responses.

## Author Contributions

YX, YF, BZ, JW, and BZ analyzed the literatures and studies and wrote the manuscript. YX and YF contributed equally to this work. All authors contributed to the article and approved the submitted version.

## Conflict of Interest

The authors declare that the research was conducted in the absence of any commercial or financial relationships that could be construed as a potential conflict of interest.
